# The Treatment of Eclamptic Convulsions

**Published:** 1912-06

**Authors:** James R. McCord

**Affiliations:** Atlanta, Ga.


### THE TREATMENT OF ECLAMPTIC CONVULSIONS.

By James R. McCord, M. D., Atlanta, Ga.

In the following brief discussion no claim is made for originality, but the treatment is all practical, and the essentials of my college and hospital teaching.

Davis says as a constant factor in all forms of eclampsia, is found some form of toxemia of longer or shorter duration.

What a warning is thrown out to us in that one brief sentence! How few doctors give to their patients that consult them soon after conception, the necessary advice as to diet, hygiene, dress and right living! How lax we are in insisting upon urine examinations! How careless we are in explaining to our patients just what regular, systematic examinations of the urine are for, and what they mean to her. How few times do we sit down and explain to her why milk is the best diet for a pregnant woman, why heavy and rare meats should be eaten so sparingly, why fruits and vegetables should be eaten so freely, why she should drink large quantities of water! Convince her that constipation is dangerous, especially if of long duration; that headache is a symptom and not a disease, and may point during pregnancy to a pathological obstetrical condition. Teach her the value of alertness, that she may tell you the slightest symptoms of approaching danger. What a simple matter it is to do these things, and what a great influence they have upon the successful completion of gestation!

The treatment of eclamptic convulsions may be divided into three classes, as to the time during pregnancy that they occur:

1. Convulsions occurring before labor.
2. Convulsions occurring at or about the time of labor.
3. Convulsions occurring just after the completion of labor.

TREATMENT OF CONVULSIONS OCCURRING BEFORE TERM.

Eclamptic attacks in the majority of instances occur in the young or rather old primiparae. The cervix is usually long and rigid. The one indication is to empty the uterus, and when this is done, the convulsions nearly always cease. In those rare cases where they do not, frequency and severity are both lessened. Any procedure that will best do this, with the least amount of shock and danger to the mother and offers the best chance to the child, is the one to use. From having seen in my hospital work a number of cases successfully handled by Vaginal Caesarean, I am convinced that it is the safest and most satisfactory method of treating these cases under this particular head. The technic in brief is as follows:

Empty the rectum by an enema, catheterize the patient on the table. Do not start the anaesthetic until everything is ready and the operator ready to begin. Ether is the anaesthetic of choice, as will be mentioned later. The patient is put in the lithotomy position and the vulva and vagina prepared as for any operation. With his hand the operator first carefully and slowly dilates the vagina. This should be done thoroughly. Depress the perineum with a speculum. The cervix is caught with tenacula and pulled into view. The operation is contraindicated when this cannot be done. With a scalpel make a circular incision at the crevico vaginal juncture. This incision is through the mucosa, and half the circumference of the cervix. With a gauze sponge strip the bladder well up out of the way and hold there with a retractor. This stripping is usually easily done because of the vascularity of the parts due to pregnancy. With a strong pair of scissors split the anterior lip of the cervix up and through the internal os. Care should be exercised in doing this to keep in the mid-line of the cervix, and if this is done there will be little or no hemorrhage. The opening made in the uterus in this way will nearly always be large enough to deliver through, but if the head of the child seems large, the posterior lip of the cervix should be split in the same way, being careful to strip back the peritoneum and the rectum. Often the slit because it admits the hand freely into the uterus is thought to be large enough, and the after coming head will cause a tear into the body of the uterus. If the operator has been careful up to this point the membranes are still intact. Rupture them, do a podalic version, and deliver. Deliver the after coming head slowly and deliberately, trying to avoid uterine and perineal tears. It may occasionally be necessary to deliver the after coming head with forceps and a pair should always be ready for immediate use. Cut the cord and hand the baby to an assistant. The placento is delivered manually. There is usually but little hemorrhage, but I believe that the loss of a considerable quantity of blood favors the prognosis. Should a post partum hemorrhage of any severity occur, the uterus should be tamponed with plain or 5% iodoform gauze. The incisions in the cervix are closed with interrupted cat-gut sutures. If the convulsions ceased, which they nearly always do, the post-operative treatment is the same as that of any other surgical case, with the exception, of course, of the diet,. This should be largely milk. Should the convulsions continue, the treatment is the same as that discussed under the third head.

TREATMENT OF CONVULSIONS OCCURRING AT OR ABOUT THE TIME OF LABOR.

Any case of eclampsia is an urgent one, and, as was stated above, the concensus of opinion at present is that the immediate emptying of the uterus offers the most favorable prognosis. The cervix is found, at this time, at different stages of dilitation. The head is sometimes in mid-pelvis and all this is necessary is to apply forceps and deliver. If cervix is soft, manual dilitation and quick delivery by forceps should be done. If this can't be done and it is thought that the cervix can be dilated within a reasonable time by bags, they may be used, delivering always at the earliest possible moment. If one of these things can't be done, delivery by Vaginal Caesarean is the best method. Rapid dilitation of a long rigid cervix by any form of instrument should never be practiced. It increases shock, causes severe lacerations that are hard to repair well, and increases the danger of sepsis and chronic invalidism. While delivering by any of the above methods, should convulsions occur, they may be controlled by veratrum viride, the use of which will be discussed under the next division.

TREATMENT OF CONVULSIONS OCCURRING JUST AFTER LABOR.

The treatment that will now be outlined is the treatment other than surgical that would be used in the conditions discussed above. The convulsions should never be controlled by chloroform. Cragin and Hull, of New York, have shown that this drug, used as an anaesthetic in puerperal toxemia and eclampsia, produces certain definite pathological changes, chiefly in the kidneys and liver. These changes are those of congestion, hemorrhage, parenchymatous degeneration, and necrosis.

Where possible, the blood pressure should be taken. It is found to be in enarly every instance high, that is, over 160 to 170. Administer at once 5 minims of the Fld. Ext. of veratrum viride by hypodermic. Note the effect by the rate and feel of the pulse, or better by the sphygmomanometer. This drug should always be given for effect and never according to its pharmaceutical dosage. If you do results will not be obtained. A good, safe rule is to give 5 minims every two hours, by hypodermic, to keep the pulse rate around 60 to 70, and the blood pressure around 140. This will usually control the convulsions. The next thing that will demand our attention is the relief of the extreme restlessness. This can be relieved nearly every time by the rectal administration of chloral hydrate, grs. xx to xxx and soda bromide drms. 2. This may be repeated every two hours as often as necessary.

The convulsions and restlessness controlled, elimination by the bowels, skin and kidneys should be attempted. Calomel in powdered form grs. 1, should be given on the tongue every hour until 8 or 10 doses have been given. High colonic irrigations of saline should be given every six hours. To stimulate the activity of the kidneys, dry cup thoroughly over the region of both kidneys, follow for 15 to 20 minutes with a mustard sinapism (1 to 4), and then place a hot water bottle over each kidney. Skin elimination should be promoted by means of the hot pack, though at times they are not indicated nor at other times necessary. A simple way of giving a hot pack is as follows:

Wrap the patient in a thick, dry blanket—another blanket is soaked in boiling hot water and then thoroughly dried by twisting with two sticks. Wrap this hot blanket around the blanket that is on the patient, care being taken to prevent burns. Around this hot blanket wrap an oil cloth, and over this another blanket. An ice bag should be put to the head, and if possible the patient should drink a glass of water. The pack should continue from 20 to 40 minutes. Hot packs are depressing and careful judgment as to patient's endurance should be exercised.

Mention only can be made of venesection and infusion as practiced by many obstetricians with good results. One or two men advocate, and have done the classical Caesarean with excellent results.

Also the use of morphia which is used routinely at the Dublin Maternity. Davis writes that we may have eclampsia, without convulsions, as manifested by some of the severe toxemias of pregnancy. No attempt has been made to discuss this phase of the subject. The writer has only in a simple way endeavored to present a plain, practical treatment for that most dreaded obstetrical complication, eclamptic convulsions.

424 Candler Bldg.

